# 25-hydroxyvitamin D deficiency, exacerbation frequency and human rhinovirus exacerbations in chronic obstructive pulmonary disease

**DOI:** 10.1186/1471-2466-12-28

**Published:** 2012-06-22

**Authors:** Jennifer K Quint, Gavin C Donaldson, Nancy Wassef, John R Hurst, Michael Thomas, Jadwiga A Wedzicha

**Affiliations:** 1 Academic Unit of Respiratory Medicine, University College London Medical School, Royal Free Campus, Rowland Hill Street, London, UK; 2 Department of Clinical Biochemistry, Royal Free Hospital, Pond Street, London, UK

## Abstract

**Background:**

25-hydroxyvitamin D deficiency is associated with COPD and increased susceptibility to infection in the general population.

**Methods:**

We investigated whether COPD patients deficient in 25-hydroxyvitamin D were more likely to be frequent exacerbators, had reduced outdoor activity and were more susceptible to human rhinovirus (HRV) exacerbations than those with insufficient and normal levels. We also investigated whether the frequency of *Fok*I, *Bsm*I and *Taq*Iα 25-hydroxyvitamin D receptor (VDR) polymorphisms differed between frequent and infrequent exacerbators.

**Results:**

There was no difference in 25-hydroxyvitamin D levels between frequent and infrequent exacerbators in the summer; medians 44.1nmol/L (29.1 – 68.0) and 39.4nmol/L (22.3 – 59.2) or winter; medians 24.9nmol/L (14.3 – 43.1) and 27.1nmol/L (19.9 – 37.6). Patients who spent less time outdoors in the 14 days prior to sampling had lower 25-hydroxyvitamin D levels (p = 0.02). Day length was independently associated with 25-hydroxyvitamin D levels (p = 0.02). There was no difference in 25-hydroxyvitamin D levels between baseline and exacerbation; medians 36.2nmol/L (IQR 22.4-59.4) and 33.3nmol/L (23.0-49.7); p = 0.43. HRV positive exacerbations were not associated with lower 25-hydroxyvitamin D levels at exacerbation than exacerbations that did not test positive for HRV; medians 30.0nmol/L (20.4 – 57.8) and 30.6nmol/L (19.4 – 48.7). There was no relationship between exacerbation frequency and any VDR polymorphisms (all p > 0.05).

**Conclusions:**

Low 25-hydroxyvitamin D levels in COPD are not associated with frequent exacerbations and do not increase susceptibility to HRV exacerbations. Independent of day length, patients who spend less time outdoors have lower 25-hydroxyvitamin D concentration.

## Background

Serum concentrations of 25-hydroxyvitamin D vary with age, race, sex, season and geographic location [1]. In its physiologically active form 25-hydroxyvitamin D is released into the circulation, binds to a carrier protein in the plasma (25-hydroxyvitamin D binding protein (DBP)) and is transported to various target organs where it mediates its biological effects by binding to the 25-hydroxyvitamin D receptor (VDR) [2]. Deficiency in 25-hydroxyvitamin D results from a number of causes and is associated with increased risk of infections including influenza, TB and pneumonia [3]. VDR dysfunction, linked to 25-hydroxyvitamin D deficiency, is thought to cause a decline in innate immune function that increases susceptibility to infections [4].

COPD is characterised by irreversible expiratory airflow limitation [5]. The disease is interspersed with periods of exacerbation that have important consequences for patients and health care providers [6-10]. Exacerbations are predominantly triggered by infection and the commonest respiratory virus detected in the airways at exacerbation is human rhinovirus (HRV) [11,12]. Some individuals are more susceptible to developing exacerbations and these individuals are termed “frequent exacerbators” [13]. Frequent exacerbators have worse quality of life [6], greater limitation of their daily activity, spend less time outdoors [14], have faster disease progression [8,15,16] and greater airway inflammation [17] and increased mortality [18] compared to patients with infrequent exacerbations.

Exacerbations are approximately 50% more likely in the winter [19]. It has been postulated that humans have improved innate immunity in the summer, impaired in the winter and that with 25-hydroxyvitamin D synthesis being so dependent on sunlight exposure, falling winter levels may trigger immune deficiencies [20]. It has also been shown recently that vitamin D deficiency is associated with increased mortality in patients admitted to hospital with community acquired pneumonia [21] and it is possible that vitamin D deficiency in COPD may increase susceptibility to pneumonia at the time of exacerbation. Genetic variants in the 25-hydroxyvitamin D pathway have been associated with chronic obstructive pulmonary disease (COPD) [22-26] and many polymorphisms in the VDR gene [27] have been linked with infection.

Our London COPD cohort, a well characterised group of patients allows us to study various aspects of 25-hydroxyvitamin D deficiency on COPD. We hypothesised that with exacerbations peaking in the winter/early spring when 25-hydroxyvitamin D levels are at their lowest, this deficiency in 25-hydroxyvitamin D may increase exacerbation risk via changes in airway inflammation. As our primary outcome we investigated whether COPD patients deficient in 25-hydroxyvitamin D were more likely to be frequent exacerbators. We also investigated whether they had reduced outdoor activity, and were more susceptible to HRV at exacerbation than those with insufficient or normal levels. We investigated whether 25-hydroxyvitamin D levels correlated with HRV load. We also investigated whether the frequency of VDR polymorphisms differed between frequent and infrequent exacerbators.

## Methods

### Patient recruitment

Ninety seven COPD patients were studied between 1^st^ April 2006 and 30^th^ March 2009. The recruitment and monitoring of patients in the London COPD cohort has previously been described [6,8,11,12,14,17]. This study was approved by the Royal Free Hospital Research Ethics Committee (Ref: 05/Q0501/126) and patients gave written informed consent.

All patients had COPD as defined by a forced expiratory volume in one second (FEV_1_) of ≤ 80% and FEV_1_ to forced vital capacity (FVC) ratio below 70% with β_2_ agonist reversibility of less than 15% or 200ml. Patients were excluded if they had other significant respiratory diseases. Patients were recruited when stable, with no exacerbations reported in the preceding month.

At the initial visit, daily respiratory symptoms, smoking history, drug history and co-morbidities were recorded. Height and weight were measured along with baseline lung function using a volumetric storage spirometer (Vitalograph 2160, Maids Moreton, Buckingham, UK). Blood was collected for 25-hydroxyvitamin D assay. Summer samples were taken in June, July or August, and winter samples in January, February or March. Both samples were collected in each individual in the same 12 month period. In each patient, spontaneously produced sputum when available and a nasopharyngeal swab (NPS) were collected for HRV detection.

### Exacerbations

COPD patients completed daily diary cards, recording any increase in daily respiratory symptoms. They were asked to contact the study team if they experienced an increase in their symptoms and were usually reviewed within 24 hours. Major symptoms were increased dyspnoea, sputum volume or sputum purulence and minor symptoms increased cough, wheeze, sore throat or coryzal symptoms. Exacerbations were defined according to our previously validated criteria of two symptoms (at least one major) for two consecutive days, or if in the opinion of the attending clinician, the patient had an exacerbation [17]. Our exacerbation definition has been validated against changes in quality of life [6], inflammatory markers [17], and FEV_1_ decline [8]. All exacerbations in this study were treated with antibiotics and steroids. None of the exacerbations required hospitalisation.

At an exacerbation visit information was collected on symptom type. The end of the exacerbation was taken as the last day on which lower airway symptoms were recorded. Spirometry was performed and blood taken for 25-hydroxyvitamin D assay. Sputum was collected if spontaneously produced and a NPS taken. All exacerbations were treated with bronchodilators, antibiotics and/or oral steroids as judged by the clinician. All samples were taken prior to the initiation of treatment. Exacerbation visits were not limited to summer or winter months and results were adjusted for seasonality.

### Exacerbation frequency

Exacerbation frequency was determined from diary cards. Patients were defined as ‘frequent exacerbators’ if they had three or more exacerbations per year, or ‘infrequent exacerbators’ if they had less than three exacerbations per year [28,29]. 3 exacerbations were chosen as both treated and untreated exacerbations were included in our definition.

### Time outdoors and daylength

At the end of each day, patients record on diary cards the number of hours they have spent out of the house that day. The time outdoors for baseline and exacerbation visits was calculated as the average time spent outdoors in the 14 days preceding the clinic visit. Daylength data were obtained from Meteorological Office data (at Heathrow Airport, London) on the day of the visit to clinic.

### Patient blood sampling

Seven millilitres of venous blood collected at baseline and exacerbation visits was centrifuged at 224 x g for 10 minutes at 4°C within two hours of collection. The serum was then separated and stored at −80°C for later analysis.

### 25-hydroxyvitamin D measurement

Samples were assayed using the LIAISON 25-OH 25-hydroxyvitamin D TOTAL (DiaSorin, Italy). The LIAISON 25-OH 25-hydroxyvitamin D TOTAL is a fully automated antibody-based two-step direct competitive chemiluminescence immunoassay (CLIA) in the clinical biochemistry department at the Royal Free Hospital. The assay recognises 100% 25-OH 25-hydroxyvitamin D2 and 25-OH 25-hydroxyvitamin D3 using magnetic micropeptide separation. The limit of detection is ≤ 4.0 ng/ml. 25-hydroxyvitamin D deficiency was defined as <25nmol/L, insufficiency 25 – 75nmol/L and sufficiency > 75nmol/L (conversion factor of 2.5 for nmol/L from ng/ml). We chose to measure 25-hydroxyvitamin D as there is a standardised assay for measurement which is widely used and previously published on. The serum concentration of 25-hydroxyvitamin D is typically used to determine vitamin D status as it reflects vitamin D produced in the skin as well as that acquired from the diet, and has a fairly long circulating half-life.

### Virus detection in NPS and sputum

Samples were collected and processed according to our previously published methodology [28]. Briefly, patients were instructed to blow their nose prior to the swab being passed gently through the nose towards the posterior nasopharynx. The swab was rotated 5–6 times and allowed to remain in place for 5 seconds. The swab was then immediately placed in an eppendorf containing 0.5ml PBS (phosphate buffered saline) and stored at −80°C until RNA extraction. Sputum samples were examined as soon as possible and within two hours of collection. The sample was separated from contaminating saliva and processed using previously published methods [30].

RNA was extracted from NPS using the High Pure Viral RNA kit (Roche) according to manufacturer instructions. RNA was extracted from sputum using Tri-reagent LS (Sigma) according to the manufacturer instructions. cDNA was prepared using the High-Capacity cDNA Reverse Transcription Kit (Applied Biosystems) following the manufacturer instructions. Real-time PCR was performed using the ABI Prism 7500 Real Time PCR System (Applied Biosystems). 25μl reaction volumes were set up (12.5μl QuantiTect Probe PCR Master Mix (ROX reference dye, Qiagen), 1μl forward and reverse primers (20μM), 0.35μl probe (20μM), 2.5μl template and 7.65μl RNase free water. PCR conditions: 95°C 15 min, 40 cycles of 95°C 15 sec and 58°C 80 sec. This methodology has been published previously [28].

### Genotyping

Venous blood samples (10-20ml) were taken in EDTA tubes at the initial visit for all patients and control subjects and stored at −80°C prior to DNA extraction. DNA extraction was performed using a Gentra® systems Puregene® genomic DNA purification kit (Qiagen Cat no. 158389) following the Whole-Blood-Enhanced Productivity protocol supplied by the manufacturer. The primers and PCR conditions are given in Table 1.

**Table 1:** Primers for VDR polymorphisms

PCR	Forward primer	Reverse primer
VDR	AGCTGGCCCTGGCACTGACTCTGCTCT	ATGGAAACACCTTGCTTCTTCTCCCTC
FokI		
VDR	GGGACGATGAGGGATGGACAGAGC	GGAAAGGGGTTAGGTTGGACAGGA
TaqI		
VDR	AACTTGCATGAGGAGGAGCATGTC	GGAGAGGAGCCTCTGTCCCATTTG
BsmI		

The specific VDR polymorphisms chosen were based on previous published literature as they are associated with functionality.

#### *Fok*I PCR

The reaction consisted of 12.5μl PCR master mix, 1.0μl Forward primer, 1.0μl Reverse primer, 8.0μl water and 2.5μl DNA. PCR machine conditions; denaturation 94°C for 5 minutes, then 40 cycles of denaturation 94°C for 30 seconds, annealing 56°C 30seconds, extension 72°C 30 seconds. The final extension was at 72°C for 5 minutes.

#### *Taq*1α PCR

The reaction consisted of 12.5μl PCR master mix, 1.0μl Forward primer, 1.0μl Reverse primer, 8.0μl water and 2.5μl DNA. PCR machine conditions; denaturation 94°C for 5 minutes, then 40 cycles of denaturation 94°C for 30 seconds, annealing 66°C 30seconds, extension 72°C 30 seconds. The final extension was at 72°C for 5 minutes.

#### *Bsm*I PCR

The reaction consisted of 12.5μl PCR master mix, 1.0μl Forward primer, 1.0μl Reverse primer, 8.0μl water and 2.5μl DNA. PCR machine conditions; denaturation 94°C for 5 minutes, then 30 cycles of denaturation 94°C for 30 seconds, annealing 56°C 30seconds, extension 72°C 30 seconds. The final extension was at 72°C for 5 minutes.

All PCR products were run on a 2% agarose gel with a PCR low ladder. The gel was run at 100mV for 15 minutes (Horizon 58, Biometra).

#### VDR *Fok*I RFLP

To genotype the samples for the *Fok*I polymorphism (rs2228570), a RFLP analysis was carried out by using the *Fok*I restriction endonuclease enzyme (R0109S New England BioLabs). The reaction consisted of; 2μl buffer, 7.5μl dH_2_O, 0.5μl RE (*Fok*I) and 10μl PCR template. The reaction digested at 37°C for 3 hours on PCR machine. The product was run on a 3% agarose gel. This resulted in the following bands; FF 265bp, Ff 265, 196 and 69bp, ff 196 and 69bp.

#### VDR *Taq*Iα RFLP

To genotype the samples for the *Taq*Iα polymorphism (rs731236), a RFLP analysis was carried out by using the *Taq*Iα restriction endonuclease enzyme (R0149T New England BioLabs). The reaction consisted of; 2μl buffer, 0.2μl BSA, 7.7μl dH_2_O, 0.1μl RE (*Taq*Iα) and 10μl PCR template. The reaction digested at 65°C for 3 hours on PCR machine. The product was run on a 3% agarose gel. This resulted in the following bands; TT 495bp, Tt 495, 290 and 205bp, tt 290 and 205bp.

#### VDR *Bsm*I RFLP

To genotype the samples for the *Bsm*I polymorphism (rs1544410), a RFLP analysis was carried out by using the *Bsm*I restriction endonuclease enzyme (R0134S New England BioLabs). The reaction consisted of; 2μl buffer, 7.75μl dH_2_O, 0.25μl RE (*Bsm*I) and 10μl PCR template. The reaction digested at 65°C for 3 hours on PCR machine. The product was run on a 3% agarose gel. This resulted in the following bands; BB 813bp, Bb 813, 670 and 145bp, bb 670 and 145bp.

### Statistical analysis

Data were analysed using SPSS version 15 or STATA 8.2 (Stat Corporation, Texas, USA). The Kolmogorov-Smirnov test of normality was applied. Normally distributed data were expressed as mean and standard deviation (SD), skewed data as median and interquartile range (IQR). Spearman rank was used to assess non-parametric correlations. Wilcoxon and Mann Whitney U tests were used for paired and unpaired non-parametric tests respectively. Adjustment for seasonality and investigation of exacerbation and baseline 25-hydroxyvitamin D levels was done using year period sine and cosine terms. Results were therefore adjusted for repeated measures. The study was powered at 0.90 at a 2-sided 0.05 significance level to detect a difference in 25-hydroxyvitamin D of 10nmol/L between frequent and infrequent exacerbators. This study was powered for the primary outcome.

## Results

### Baseline patient characteristics

Ninety seven COPD patients were studied, 61 male and 36 female. The patients had a mean FEV_1_ of 1.19 l or 50.3% predicted. Ten COPD patients were on 25-hydroxyvitamin D supplementation (Calcichew D3), and were not included in the following analysis unless otherwise stated. Each tablet of calcichew D3 contains 10 micrograms of colecalciferol and patients usually take 2 a day. The baseline characteristics are reported in Table 2.

**Table 2:** Baseline Characteristics of 97 patients

	COPD patients (n = 97) Mean (SD)
Age (years)	71.8 (8.8)
FEV1 (litre)	1.19 (0.54)
FEV1 (% predicted)	50.3 (19.7)
FVC (litre)	2.5 (0.84)
BMI (kgm^-2^)	27.0 (6.0)
Pack years smoking	50.7 (34.2)
SpO_2_ (%) on air	95 (2)
25-hydroxyvitamin D nmol/L	Median (IQR)
Summer	41.3 (26.8 – 64.8)
Winter	27.8 (19.4 – 44.4)
	Number (%)
Male	61 (62.9)
Frequent exacerbators	28 (28.9)
Current smokers	25 (25.8)

### Seasonal variation and 25-hydroxyvitamin D levels in COPD

**Figure 1:**
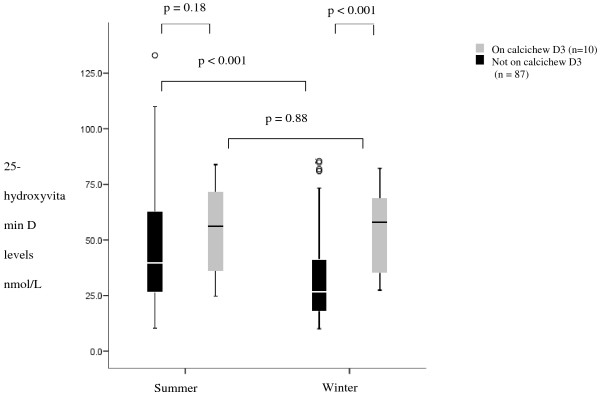
**25-hydroxyvitamin D levels in COPD patients in summer and winter in those on and not on calcium and 25-hydroxyvitamin D supplementation.** Data are presented as median, with the boxes representing the interquartile range and the whiskers representing SD. ○: extreme outliers.

COPD patients had lower 25-hydroxyvitamin D levels in winter compared to summer; medians 26.7nmol/L (IQR 17.8 – 41.2) and 39.6nmol/L (26.4 – 62.9); p < 0.001. Within individuals, summer and winter 25-hydroxyvitamin D levels varied by 10% monthly and by 50% between summer and winter, with summer levels being higher. Patients taking Calcichew D3 did not show the same significant seasonal variation in 25-hydroxyvitamin D levels; winter median 58.1nmol/L (35.2 – 69.6), summer median 56.2nmol/L (33.9 – 73.1; p > 0.05. The seasonal difference in 25-hydroxyvitamin D levels between these patients is illustrated in Figure 1. There were no differences in summer 25-hydroxyvitamin D levels between current smokers and ex-smokers, medians; 45.5nmol/L (27.4 – 63.3) and 40.3 nmol/L (26.7 – 66.3) or winter 25-hydroxyvitamin D levels; medians 27.0 nmol/L (14.6 – 47.7) and 28.0 nmol/L (19.9 – 43.7).

### 25-hydroxyvitamin D levels in frequent and infrequent exacerbators

**Figure 2:**
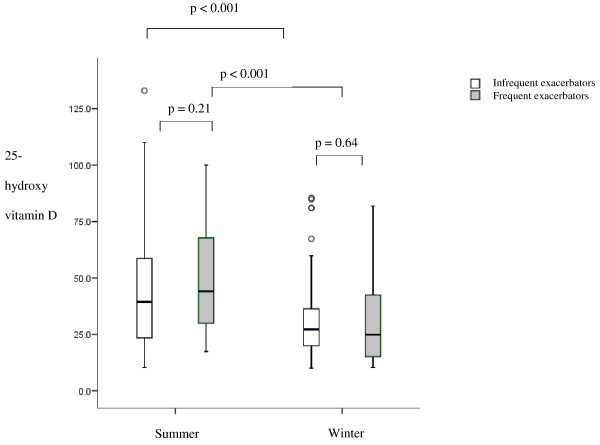
**25-hydroxyvitamin D levels in summer and winter in frequent and infrequent exacerbators.** Data are presented as median, with the boxes representing the interquartile range and the whiskers representing SD. ○: extreme outliers.

Figure 2 shows that there was no difference in 25-hydroxyvitamin D levels between frequent exacerbators (1/3 of the cohort) and infrequent exacerbators in the summer; medians 44.1nmol/L (29.1 – 68.0) and 39.4nmol/L (22.3 – 59.2) or winter; medians 24.9nmol/L (14.3 – 43.1) and 27.1nmol/L (19.9 – 37.6). The proportion of patients’ deficient, insufficient and sufficient in 25-hydroxyvitamin D was the same in both frequent and infrequent exacerbators groups. Exacerbation history was available in a subset of 10 patients before and after treatment with Calcichew D3. There was no difference in actual exacerbation number from year 1 to 2; p = 0.45, or in exacerbation frequency from year 1 to 2; p = 0.38.

### Time outdoors and 25-hydroxyvitamin D levels

**Figure 3:**
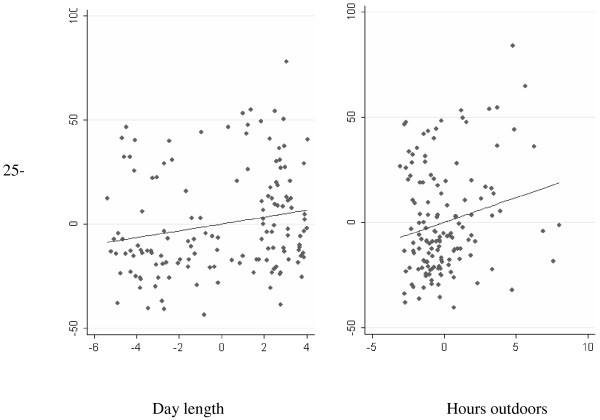
**Adjusted partial residual plot of the relationship between 25-hydroxyvitamin D levels and (1) day length (hours), and (2) hours outdoors.** The graphs show the effect of each variable on 25-hydroxyvitamin D independently of the other variable. The zero value is the annual average.

Figure 3 shows that shorter day length on the day of sampling was associated with lower levels of 25-hydroxyvitamin D (coef 1.65, se 0.69; p = 0.02). Patients who spent less time outdoors in the 14 days prior to sampling also had lower 25-hydroxyvitamin D levels coef 2.36, se0.96; p = 0.02). This was independent of day length.

### 25-hydroxyvitamin D levels and exacerbations of COPD

We measured serum 25-hydroxyvitamin D levels in 58 exacerbations; 1 per patient in the study period. Adjusting for seasonality, there was no difference in 25-hydroxyvitamin D levels between baseline and exacerbation; medians 36.2nmol/L (IQR 22.4-59.4) and 33.3nmol/L (23.0-49.7); p = 0.43.

### 25-hydroxyvitamin D and HRV exacerbations

46 exacerbations were tested for the presence of HRV in sputum or NPS. HRV positive exacerbations (n = 12, viral load > 175pfu/ml; [28] were not associated with lower 25-hydroxyvitamin D levels at exacerbation than exacerbations that did not test positive for HRV; medians 30.0nmol/L (20.4 – 57.8) and 30.6nmol/L (19.4 – 48.7). HRV load in sputum or NPS at exacerbation did not correlate with exacerbation 25-hydroxyvitamin D levels (data not shown). When including all exacerbations per patient taken over the study period tested for HRV, patients deficient in 25-hydroxyvitamin D at baseline did not have an increased proportion of HRV positive exacerbations compared to those insufficient or sufficient. This is illustrated in Table 3. The presence of cold symptoms at exacerbation was not related to vitamin D deficiency (p > 0.05).

**Table 3:** 25-hydroxyvitamin D status and positivity for HRV at exacerbation

25-hydroxyvitamin	positive for HRV at exacerbation		Total
D status	No	Yes	
Deficient	4 (25.0%)	7 (23.3%)	11
Insufficient	10 (62.5%)	19 (63.3%)	29
Sufficient	2 (12.5%)	4 (13.3%)	6
Total	16	30	46

25-hydroxyvitamin D deficiency was defined as <25nmol/L, insufficiency 25 – 75nmol/L and sufficiency > 75nmol/L (conversion factor of 2.5 for nmol/L from ng/ml).

### Exacerbation length and severity

There was no relationship with 25-hydroxyvitamin D levels either at that exacerbation or the baseline preceding that exacerbation and the length of the exacerbation; rho = −0.41: p = 0.12 and rho = −0.41: p = 0.12 respectively or time to the next exacerbation; rho = −0.11, p = 0.66 and rho = −0.06, p = 0.81 respectively. All exacerbations recovered.

### VDR polymorphisms

All genotypes were within Hardy Weinberg equilibrium. The *Fok*I polymorphism was not related to *Taq*I or *Bsm*I, however the *Bsm*I and *Taq*I polymorphisms were linked (p < 0.001). There was no relationship with genotyping and exacerbation frequency for any of the polymorphisms as illustrated in Table 4.

**Table 4:** VDR polymorphisms and exacerbation frequency

SNP	Genotype	Frequent exacerbators (n = 28)	Infrequent exacerbators (n = 68)	Chi squared P value
Rs1544410	BB	3 (10.7%)	15 (22.1%)	
BsmI	Bb	12 (42.9%)	26 (38.2%)	0.43
	Bb	13 (46.4%)	27 (39.7%)	
HWE p value		0.93	0.08	
		(n = 26)	(n = 66)	
Rs731236	TT	10 (38.5%)	24 (36.4%)	
TaqI	Tt	13 (50%)	29 (43.9%)	0.64
	Tt	3 (11.5%)	13 (19.7%)	
HWE p value		0.69	0.43	
		(n = 28)	(n = 68)	
Rs2228570	FF	10 (35.7%)	21 (30.9%)	
FokI	Ff	14 (50.0%)	38 (55.9%)	0.87
	Ff	4 (14.3%)	9 (13.2%)	
HWE p value		0.80	0.21	

## Discussion

We have investigated several aspects of 25-hydroxyvitamin D deficiency in a well characterised cohort of COPD patients and shown that independent of day length, COPD patients who spend less time outdoors have lower 25-hydroxyvitamin D levels. In this study we found that patients on Calcichew D3 had much higher levels of 25-hydroxyvitamin D than COPD patients not on 25-hydroxyvitamin D supplementation and showed much less seasonal variation in 25-hydroxyvitamin D levels. This is an interesting finding as several studies have investigated the pharmacokinetics of 25-hydroxyvitamin D, and it is thought that the amount of cholecalciferol in Calcichew is far below the amount required to treat deficiency and maintain a stable 25-hydroxyvitamin D level. This suggests that perhaps these patients may differ with respect to underlying sub-phenotype or co-morbidities (e.g. osteoporosis). However with only 10 patients in this group it is not possible to reach firm conclusions.

### Outdoor activity and 25-hydroxyvitamin D levels

From this study it appears that 25-hydroxyvitamin D levels are driven primarily by season and outdoor activity, with patients with lower 25-hydroxyvitamin D levels spending less time outdoors in the 14 days preceding sampling. This has implications for pulmonary rehabilitation, patient education and lifestyle modification. Independent of 25-hydroxyvitamin D status, reduced activity in COPD is related to poorer prognosis [31]. Perhaps outdoor pulmonary rehabilitation should be encouraged in those COPD patients deficient in 25-hydroxyvitamin D.

A large proportion of the elderly population in the USA and Europe are 25-hydroxyvitamin D deficient [32] and those with COPD are at particularly high risk [1]. Studies in asthma linking low 25-hydroxyvitamin D levels with disease severity have postulated the relationship may be secondary to time spent indoors [33]. This is also likely to be important in COPD [14]. 25-hydroxyvitamin D deficiency in the COPD population may reflect poor general health status rather than having particular importance in the natural progression of COPD. However, if it were that simple, patients with COPD living in tropical countries should have milder disease and fewer exacerbations.

### 25-hydroxyvitamin D levels and exacerbations and exacerbation frequency

Patients deficient in 25-hydroxyvitamin D in the stable state (< 25nmol/L), were not more likely to be frequent exacerbators and did not have a shorter time to their next exacerbation than those insufficient or sufficient in 25-hydroxyvitamin D. 25-hydroxyvitamin D levels were unchanged between baseline and exacerbation in COPD when adjustments were made for seasonality and we did not find a relationship between 25-hydroxyvitamin D deficiency and exacerbation severity. This is in keeping with a recent study which found that low baseline 25-hydroxyvitamin D levels in patients with severe COPD did not predict subsequent exacerbations [34]. A recent RCT has also shown that high dose vitamin D supplementation in COPD patients did not reduce exacerbation incidence [35]. However the authors of this study in a post-hoc analysis suggested that in patients with the most severe vitamin D deficiency at baseline, supplementation may reduce future exacerbations.

Influenza and other viruses show a distinct predilection for wintertime infectivity, and exacerbations of COPD are significantly more likely to occur in the winter. 25-hydroxyvitamin D deficiency has been associated with self-reported upper respiratory tract infections (URTI) [36,37]. As HRV is the commonest cause of colds and present in over 50% of COPD exacerbations, we chose specifically to investigate a relationship between HRV exacerbations and 25-hydroxyvitamin D deficiency. We did not find a relationship between 25-hydroxyvitamin D deficiency and the presence of HRV at exacerbation or HRV load at exacerbation. In other diseases, 25-hydroxyvitamin D supplementation studies have shown mixed results with regards to preventing viral and bacterial infections [38,39]. We did not have bacterial culture data available in this study and were unable to investigate any association between vitamin D levels and bacterial exacerbations.

### VDR polymorphisms

We did not find a relationship between VDR polymorphisms and exacerbation frequency. However, the FokI, and TaqI Vitamin D receptor polymorphisms have been shown to be associated with lower respiratory tract infections in children [40]. Genetic variants in the 25-hydroxyvitamin D pathway have been associated with COPD [1,24]. Although GWAS studies did not find VDR to be an important risk gene for COPD, associations of VDR with risk for infections [40] has been found in investigating interactions between low 25-hydroxyvitamin D levels and the VDR. Many polymorphisms exist in the VDR gene and the influence of these polymorphisms on VDR protein function may influence immunomodulatory responses [27]. To date no study has found a link with VDR polymorphisms and airway infection in COPD although there are several mechanisms by which activated 25-hydroxyvitamin D binding to the VDR could modulate viral lower respiratory tract disease [41-43]. Our study was powered on the primary outcome and our sample size for a genetic study is relatively small. This may explain our negative findings.

There are several strengths to this study. The London COPD cohort is a well characterised cohort of COPD patients with detailed information on exacerbations and exacerbation frequency. In this cohort monitoring visits are predefined at regular time intervals and driven by clinical visits because of exacerbations thus allowing information to be obtained on 25-hydroxyvitamin D at baseline and exacerbation.

We do not actually know what constitutes 25-hydroxyvitamin D deficiency, particularly in the context of its immunomodulatory properties. In terms of calcaemic effects, levels below 50nmol/L are probably deficient [3,44]. With regards to the immunomodulatory mechanisms of 25-hydroxyvitamin D it has even been suggested that levels > 100nmol/L are needed for optimal immune functioning. There is much to be learned about the role of 25-hydroxyvitamin D in COPD and the mechanisms by which increasing 25-hydroxyvitamin D levels into the normal range would influence the natural history of COPD.

## Conclusions

In conclusion, we have shown that independent of day length, patients who spend less time outdoors have lower 25-hydroxyvitamin D levels. Low 25-hydroxyvitamin D levels in COPD are not associated with exacerbation frequency and do increase susceptibility to HRV exacerbations.

## Competing interests

The authors declare that they have no competing interests.

## Authors’ contributions

JKQ, GCD, JRH, NW, MT and JAW contributed to the conception and design of the study. JKQ, to the acquisition of samples, NW to vitamin D processing, JKQ and GCD to data analysis, JKQ, GCD, JRH, NW, MT and JAW to interpretation of data. JKQ wrote the first draft of the manuscript and all authors contributed to subsequent drafts. All authors read and approved the final manuscript.

## Pre-publication history

The pre-publication history for this paper can be accessed here:

http://www.biomedcentral.com/1471-2466/12/28/prepub

## References

[B1] Janssens W Lehouck A Carremans C Bouillon R Mathieu C Decramer M 25-hydroxyvitamin D beyond bones in chronic obstructive pulmonary disease: time to act Am J Respir Crit Care Med 2009 179 8 630 636 10.1164/rccm.200810-1576PP 19164701

[B2] Mora JR Iwata M von Andrian UH Vitmain effects on the immune system: vitamins A and D take centre stage Nature Reviews Immunol 2008 8 685 698 10.1038/nri2378 PMC2906676 19172691

[B3] Holik MF 25-hydroxyvitamin D deficiency N Eng J Med 2007 357 266 281 10.1056/NEJMra070553

[B4] Waterhouse JC Perez TH Albert PJ Reversing bacteria-induced 25-hydroxyvitamin D receptor dysfunction is key to autoimmune disease Ann N Y Acad Sci 2009 1173 757 765 10.1111/j.1749-6632.2009.04637.x 19758226

[B5] Global Initiative for Chronic Obstructive Lung Disease Global Strategy for the diagnosis, management and prevention of chronic obstructive pulmonary disease. (GOLD) 2006 , http://www.goldcopd.org. Accessed 17th May 2009

[B6] Seemungal TA Donaldson GC Paul EA Bestall JC Jeffries DJ Wedzicha JA Effect of exacerbation on quality of life in patients with chronic obstructive pulmonary disease Am J Respir Crit Care Med 1998 157 1418 1422 9603117 10.1164/ajrccm.157.5.9709032

[B7] Spencer S Jones PW Time course of recovery of health status following an infective exacerbation of chronic bronchitis Thorax 2003 58 589 593 10.1136/thorax.58.7.589 12832673 PMC1746751

[B8] Donaldson GC Seemungal TAR Bhowmik A Wedzicha JA Relationship between exacerbation frequency and lung function decline in chronic obstructive pulmonary disease Thorax 2002 57 847 852 10.1136/thorax.57.10.847 12324669 PMC1746193

[B9] O’Brien JA Ward AJ Jones MKC McMillan C Lordan N Utilization of health care services by patients with chronic obstructive pulmonary disease Respir Med 2003 97 1 S53 S58 12564611 10.1016/s0954-6111(03)80015-x

[B10] Groenewegen KH Schols AMWJ Wouters EFM Mortality and mortality related factors after hospitalisation for acute exacerbation of COPD Chest 2003 124 459 467 10.1378/chest.124.2.459 12907529

[B11] Seemungal TAR Harper-Owen R Bhowmik A Respiratory viruses, symptoms and inflammatory markers in acute exacerbations and stable chronic obstructive pulmonary disease Am J Respir Crit Care Med 2001 164 1618 1623 11719299 10.1164/ajrccm.164.9.2105011

[B12] Wilkinson TMA Hurst JR Perera WR Effect of interactions between lower airway bacterial and rhinoviral infection in exacerbations of COPD Chest 2006 129 317 324 10.1378/chest.129.2.317 16478847 PMC7094441

[B13] Hurst JR Vestbo J Anzueto A Locantore N Müllerova H Tal-Singer R Miller B Lomas DA Agusti A Macnee W Calverley P Rennard S Wouters EF Wedzicha JA Evaluation of COPD Longitudinally to Identify Predictive Surrogate Endpoints (ECLIPSE) Investigators. Susceptibility to exacerbation in chronic obstructive pulmonary disease N Engl J Med 2010 363 12 1128 1138 10.1056/NEJMoa0909883 20843247

[B14] Donaldson GC Wilkinson TM Hurst JR Perera WR Wedzicha JA Exacerbations and time spent outdoors in chronic obstructive pulmonary disease Am J Respir Crit Care Med 2005 171 446 452 15579723 10.1164/rccm.200408-1054OC

[B15] Kanner RE Anthonisen NR Connett JE Lower respiratory illnesses promote FEV1 decline in current smokers but not ex-smokers with mild chronic obstructive pulmonary disease Am J Respir Crit Care Med 2001 164 358 364 11500333 10.1164/ajrccm.164.3.2010017

[B16] Celli BR Thomas NE Anderson JA Ferguson GT Jenkins CR Jones PW Vestbo J Knobil K Yates JC Calverley PMA Effect of Pharmacotherapy on Rate of Decline of Lung Function in Chronic Obstructive Pulmonary Disease: Results from the TORCH Study Am J Respir Crit Care Med 2008 178 332 338 10.1164/rccm.200712-1869OC 18511702

[B17] Bhowmik A Seemungal TAR Sapsford RJ Wedzicha JA Relation of sputum inflammatory markers to symptoms and lung function changes in COPD exacerbations Thorax 2000 55 114 120 10.1136/thorax.55.2.114 10639527 PMC1745686

[B18] Soler-Cataluna JJ Martinez-Garcia MA Roman Sanchez P Salcedo E Navarro M Ochando R Severe acute exacerbations and mortality in patients with chronic obstructive pulmonary disease Thorax 2005 57 137 141 10.1136/thx.2005.040527 PMC1747235 16055622

[B19] Eccles R An Explanation for the Seasonality of Acute Upper Respiratory Tract Viral Infections Acta Otolaryngol 2002 122 2 183 191 10.1080/00016480252814207 11936911

[B20] Hope-Simpson RE The role of season in the epidemiology of influenza J Hyg (Lond) 1981 86 1 35 47 10.1017/S0022172400068728 7462597 PMC2134066

[B21] Leow L Simpson T Cursons R Karalus N Hancox RJ Vitamin D, innate immunity and outcomes in community acquired pneumonia Respirology 2011 16 4) 611 616 21244571 10.1111/j.1440-1843.2011.01924.x

[B22] Janssens W Bouillon R Claes B Carremans C Lehouck A Buysschaert I Coolen J Mathieu C Decramer M Lambrechts D 25-hydroxyvitamin D deficiency is highly prevalent in COPD and correlates with variants in the 25-hydroxyvitamin D-binding gene Thorax 2010 65 3 215 220 10.1136/thx.2009.120659 19996341

[B23] Forli L Bjortuft O Boe J Vitamin D status in relation to nutritional depletion and muscle function in patients with advanced pulmonary disease Exp Lung Res 2009 Aug 35 6 524 538 10.1080/01902140902763193 19842836

[B24] Schellenberg D Pare PD Weir TD Spinelli JJ Walker BAM Sandford AJ 25-hydroxyvitamin D Binding Protein Variants and the Risk of COPD Am J Respir Crit Care Med 1998 157 957 961 9517617 10.1164/ajrccm.157.3.9706106

[B25] Lauridsen AL Vestergaard P Plasma concentrations of 25-Hydroxy-25-hydroxyvitamin D and 1,25-Dihydroxy-25-hydroxyvitamin D are Related to the Phenotype of Gc (25-hydroxyvitamin D-Binding Protein): A Cross-sectional Study on 595 Early Postmenopausal Women Calcified Tissue International, Springer New York 2005 77 1 15 22 10.1007/s00223-004-0227-5 15868280

[B26] Taes YEC Goemaere S Huang G Van Pottelbergh I De Bacquer D Verhasselt B Van den Broeke C Delanghe JR Kaufman JM 25-hydroxyvitamin D binding protein, bone status and body composition in community-dwelling elderly men Bone 2006 38 5 701 707 10.1016/j.bone.2005.10.006 16309986

[B27] Uitterlinden AG Fang Y Van Meurs JB Pols HA Van Leeuwen JP Genetics and biology of 25-hydroxyvitamin D receptor polymorphisms Gene 2004 338 2 143 156 10.1016/j.gene.2004.05.014 15315818

[B28] Quint JK Donaldson GC Goldring JJ Baghai-Ravary R Hurst JR Wedzicha JA Serum IP-10 as a biomarker of human rhinovirus infection at exacerbation of COPD Chest 2010 Apr 137 4 812 822 10.1378/chest.09-1541 19837822 PMC2851557

[B29] Garcia-Aymerich J Lange P Benet M Schnohr P Antó JM Regular physical activity reduces hospital admission and mortality in chronic obstructive pulmonary disease: a population based cohort study Thorax 2006 61 9 772 778 10.1136/thx.2006.060145 16738033 PMC2117100

[B30] Chapuy MC Preziosi P Maamer M Arnaud S Galan P Hercberg S Meunier PJ Prevalence of 25-hydroxyvitamin D insufficiency in an adult normal population Osteoporos Int 1997 7 5 439 443 10.1007/s001980050030 9425501

[B31] Brehm JM Celedón JC Soto-Quiros ME Avila L Hunninghake GM Forno E Laskey D Sylvia JS Hollis BW Weiss ST Litonjua AA Serum 25-hydroxyvitamin D levels and markers of severity of childhood asthma in Costa Rica Am J Respir Crit Care Med 2009 179 9 765 771 10.1164/rccm.200808-1361OC 19179486 PMC2675563

[B32] Kunisaki KM Niewoehner DE Connett JE COPD Clinical Research Network, Vitamin D levels and risk of acute exacerbations of chronic obstructive pulmonary disease: a prospective cohort study Am J Respir Crit Care Med 2012 Feb 1 185 3 286 290 10.1164/rccm.201109-1644OC 22077070 PMC3297108

[B33] Lehouck A Mathieu C Carremans C Baeke F Verhaegen J Van Eldere J Decallonne B Bouillon R Decramer M Janssens W High doses of vitamin D to reduce exacerbations in chronic obstructive pulmonary disease: a randomized trial Ann Intern Med 2012 Jan 17 156 2 105 114 22250141 10.7326/0003-4819-156-2-201201170-00004

[B34] Ginde AA Mansbach JM Camargo CA Association between serum 25-hydroxy25-hydroxyvitamin D level and upper respiratory tract infection in the Third National Health and Nutrition Examination Survey Arch Intern Med 2009 169 4 384 390 10.1001/archinternmed.2008.560 19237723 PMC3447082

[B35] Laaksi I Ruohola JP Tuohimaa P Auvinen A Haataja R Pihlajamäki H Ylikomi T An association of serum 25-hydroxyvitamin D concentrations < 40 nmol/L with acute respiratory tract infection in young Finnish men Am J Clin Nutr 2007 86 3 714 717 17823437 10.1093/ajcn/86.3.714

[B36] Martineau AR Wilkinson RJ Wilkinson KA Newton SM Kampmann B Hall BM Packe GE Davidson RN Eldridge SM Maunsell ZJ Rainbow SJ Berry JL Griffiths CJ A Single Dose of 25-hydroxyvitamin D Enhances Immunity to Mycobacteria Am J. Respir. Crit. Care Med 2007 176 208 213 10.1164/rccm.200701-007OC 17463418

[B37] Wejse C Gomes VF Rabna P Gustafson P Aaby P Lisse IM Andersen PL Glerup H Sodemann M 25-hydroxyvitamin D as Supplementary Treatment for Tuberculosis: A Double-blind, Randomized, Placebo-controlled Trial Am J Respir Crit Care Med 2009 179 843 850 10.1164/rccm.200804-567OC 19179490

[B38] Roth DE Jones AB Prosser C Robinson JL Vohra S 25-hydroxyvitamin D receptor polymorphisms and the risk of acute lower respiratory tract infection in early childhood J Infect Dis 2008 197 5 676 680 10.1086/527488 18266602

[B39] Sadeghi K Wessner B Laggner U 25-hydroxyvitamin D3 down-regulates monocyte TLR expression and triggers hyporesponsiveness to pathogen associated molecular patterns Eur J Immunol 2006 36 361 370 10.1002/eji.200425995 16402404

[B40] Bhalla AK Amento EP Krane SM Differential effects of 1,25 dihydroxy25-hydroxyvitamin D3 on human lymphocytes and monocyte/macrophages: inhibition of interleukin 2 and augmentation of interleukin1 production Cell Immunol 1986 98 311 322 10.1016/0008-8749(86)90291-1 3489547

[B41] Liu PT Stenger S Li H Toll-like receptor triggering of a 25-hydroxyvitamin D–mediated human antimicrobial response Science 2006 311 1770 1773 10.1126/science.1123933 16497887

[B42] Norman AW Bouillon R Whiting SJ Vieth R Lips P 13th Workshop consensus for 25-hydroxyvitamin D nutritional guidelines The Journal of Steroid Biochemistry and Mol Biol 2007 103 3–5 204 205 10.1016/j.jsbmb.2006.12.071 PMC1906737 17234402

[B43] Quint JK Donaldson GC Hurst JR Goldring JJ Seemungal TR Wedzicha JA Predictive accuracy of patient reported exacerbation frequency in chronic obstructive pulmonary disease Eur Respir J 2011 37 3 501 507 10.1183/09031936.00035909 20650988

[B44] Bhowmik A Seemungal TAR Sapsford RJ Devalia JL Wedzicha JA Comparison of spontaneous and induced sputum for investigation of airway inflammation in chronic obstructive pulmonary disease Thorax 1998 53 953 956 10.1136/thx.53.11.953 10193394 PMC1745116

